# Destabilizing the genome as a therapeutic strategy to enhance response to immune checkpoint blockade: a systematic review of clinical trials evidence from solid and hematological tumors

**DOI:** 10.3389/fphar.2023.1280591

**Published:** 2024-01-09

**Authors:** Faizah Alotaibi, Kanaan Alshammari, Badi A. Alotaibi, Hashem Alsaab

**Affiliations:** ^1^ College of Science and Health Professions, King Saud Bin Abdulaziz University for Health Sciences, Alahsa, Saudi Arabia; ^2^ King Abdullah International Medical Research Center, Ministry of National Guard-Health Affairs, Riyadh, Saudi Arabia; ^3^ Oncology Department, King Abdulaziz Medical City, Ministry of National Guard-Health Affairs, Riyadh, Saudi Arabia; ^4^ College of Medicine, King Saud Bin Abdulaziz University for Health Sciences, Riyadh, Saudi Arabia; ^5^ Department of Clinical Laboratory Sciences, College of Applied Medical Sciences, King Saud Bin Abdulaziz University for Health Sciences, Riyadh, Saudi Arabia; ^6^ Department of Pharmaceutics and Pharmaceutical Technology, Taif University, Taif, Saudi Arabia

**Keywords:** ICI, chemotherapies, clinical trail, cancer, radiotherapy, destabilizing the genome, immune checkpoint inhibition

## Abstract

**Background:** Genomic instability is increased alterations in the genome during cell division and is common among most cancer cells. Genome instability enhances the risk of initial carcinogenic transformation, generating new clones of tumor cells, and increases tumor heterogeneity. Although genome instability contributes to malignancy, it is also an *“Achilles’ heel”* that constitutes a therapeutically-exploitable weakness—when sufficiently advanced, it can intrinsically reduce tumor cell survival by creating DNA damage and mutation events that overwhelm the capacity of cancer cells to repair those lesions. Furthermore, it can contribute to extrinsic survival-reducing events by generating mutations that encode new immunogenic antigens capable of being recognized by the immune system, particularly when anti-tumor immunity is boosted by immunotherapy drugs. Here, we describe how genome-destabilization can induce immune activation in cancer patients and systematically review the induction of genome instability exploited clinically, in combination with immune checkpoint blockade.

**Methods:** We performed a systematic review of clinical trials that exploited the combination approach to successfully treat cancers patients. We systematically searched PubMed, Cochrane Central Register of Controlled Trials, Clinicaltrials.gov, and publication from the reference list of related articles. The most relevant inclusion criteria were peer-reviewed clinical trials published in English.

**Results:** We identified 1,490 studies, among those 164 were clinical trials. A total of 37 clinical trials satisfied the inclusion criteria and were included in the study. The main outcome measurements were overall survival and progression-free survival. The majority of the clinical trials (30 out of 37) showed a significant improvement in patient outcome.

**Conclusion:** The majority of the included clinical trials reported the efficacy of the concept of targeting DNA repair pathway, in combination with immune checkpoint inhibitors, to create a *“ring of synergy”* to treat cancer with rational combinations.

## 1 Introduction

Genomic instability is one of the hallmarks of cancer (Hanahan, 2022). Increased genomic instability can lead to increases risk of tumorgenicity and tumor heterogeneity, which could contribute to poor patient outcome (Junttila and de Sauvage, 2013). Maintaining genomic integrity is critical to prevent DNA lesions caused by chemical, physical or physiological triggers such as chemical agents, ultraviolet light or ionizing radiation (Jackson and Bartek, 2009). Mutations in genes that regulate mechanisms involved in DNA synthesis and repair in addition to loss or gain of gene function such of tumor suppressor genes and oncogenes, respectively, can cause genomic instability. From a structural level, genomic instability can occur at small DNA structures such as mutations in base pairs and microsatellites (repetitive DNA motifs) or at significant structural level such as changes in chromosomal number and structure (Petropoulos et al., 2019).

**FIGURE 1:**
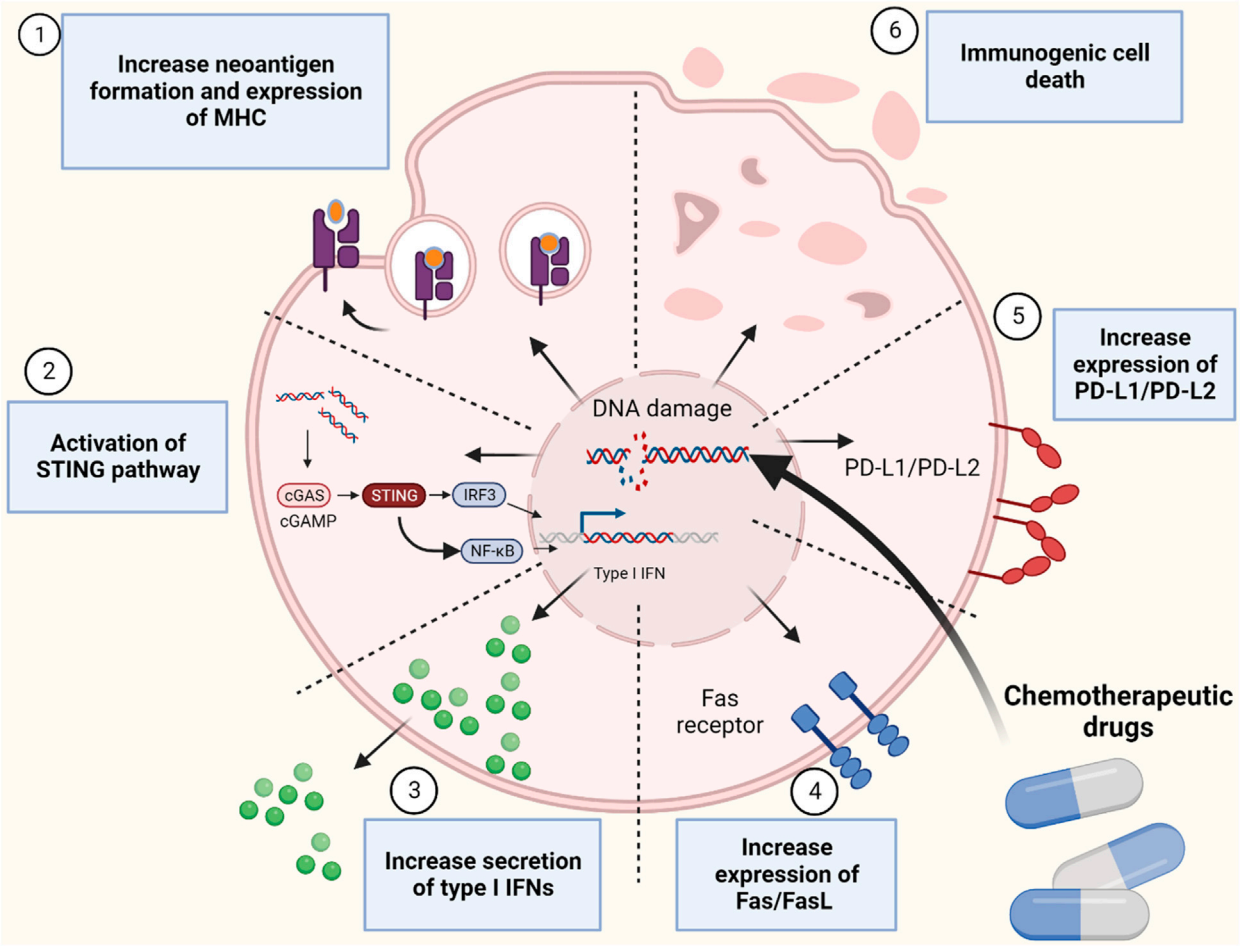
Mechanismes of immune system activation by inducing genomic instability using chemotherapeutic drugs. Figure is generated using Biorender.com.

Genomic integrity is maintained by surveillance mechanisms known as DNA damage response (DDR) (Sirbu and Cortez, 2013). Chemotherapy drugs can overwhelm tumor cells by creating more DNA mutations and damages that will work as “*Achilles’ heels*”. These drugs could generate defects in DDR system, including DNA damage sensor and protein kinases that facilitate the repair of DNA lesions resulting in the accumulation of catastrophic DNA lesions leading to tumor cell apoptosis (Pilié et al., 2019). Moreover, depending on the type of chemotherapeutic drugs, they could also activate immune system by inducing immunological tumor death and activating signaling pathways that promote adaptive immune response (Fabian et al., 2021) (Figure 1). Previously, anti-cancer chemotherapy drugs were tested on immunocompromised animals (Taetle et al., 1987). Therefore, the role of immune cells in chemo-treated mice is not well understood. With the recent interests in harnessing the power of the immune system in oncology, increasing number of studies have investigated the effect of various chemotherapeutic drugs in immunocompetent mice and found it to be more effective in tumor killing (Ottewell et al., 2012). This suggest that activation of the immune system can be a secondary effect of some chemotherapy drugs. These drugs can activate both the innate and adaptive immune system by direct and indirect effects on immune cells (Hartl et al., 2019). Indirect effect by promoting cellular rearrangements that help tumor recognition by immune cells and directly by inducing transient lymphodepletion of immunosuppressive immune cells such as myeloid-derived suppressor cells (MDSCs) (Wang et al., 2017a). All these evidence suggest that immunomodulatory chemotherapeutic drugs can be good candidates for adjuvant chemotherapy in combination with cancer immunotherapy such as immune checkpoint inhibitors (ICIs). In this work, we will discuss mechanisms of which DNA-damaging agents can induce immune cell activation, we will explore this effect on sensitizing tumor cells to ICIs and finally explore the clinical trails that used this strategy.

## 2 Materials and methods

For the first part of this manuscript, we performed search on the preclinical studies to illustrate the principal of targeting DNA to induce response to immune checkpoint blockade. For the second part, we conducted a systematic review on the clinical trials testing these rational combinations. The Preferred Reporting Items for Systematic Reviews and Meta-Analyses (PRISMA) guidelines was used (Page et al., 2021a; Page et al., 2021b). Eligible criteria include clinical trials published in English evaluating the efficacy of any type of chemotherapies or radiation in combination with any of the eleven ICIs (ipilimumab, pembrolizumab, nivolumab, cemiplimab, camrelizumab, sintilimab, Sugemalimab, toripalimab, atezolizumab, durvalumab, and avelumab). Studies evaluating the impact of combining DNA repair inhibitors and ICI studies other than clinical trials were excluded. In addition, clinical trials evaluating tyrosine kinase inhibitors with ICIs and DNA repairs inhibitors were also excluded. We did not restrict the search to specific cancer types. Databases (PubMed, Cochrane Central Register of Controlled Trials and Clinicaltrials.gov) as well as other publication from the reference list of related articles were used for the search. Search were intended to identify all clinical trials testing this approach with any of the following key work search: chemotherapies (MeSH term) and immune checkpoint blockade (MeSH term). The systematic search did not use date restrictions and included all clinical trials published till October 2023. Meta analysis was not conducted due to the heterogeneity in the study drugs, design and cancer types. Based on the initial search, a total of 1,490 studies were found. Of these, only 164 were identified as clinical trials which were screened for the inclusion criteria. Among the 164, 102 studies were determined to be ineligible due to out of the scope drug design (Cell therapy, tyrosine kinase inhibitors, chemotherapies in cancer expressing immune checkpoint molecules, combination of ICI in High MSI cancer, biomarker studies). Ten trials were removed owning to different study design, four trials were not relevant, and 11 trials were still recruiting. Thus, 27 clinical trials and ten from reference list from relevant studies were found to be eligible to be incorporated into the systematic review. The Rayyan website were used to screen the retrieved clinical trials. The risk of bias was assessed using Cochrane risk of bias assessment tool (Higgins and Green, 2008). The tool contains main domains to assess risk of bias in appropriateness of study design, methods, measurements, data reporting and funding bias. Each domain is classified as low, unclear or high risk of bias.

## 3 Major mechanisms to prevent genome instability

**FIGURE 2:**
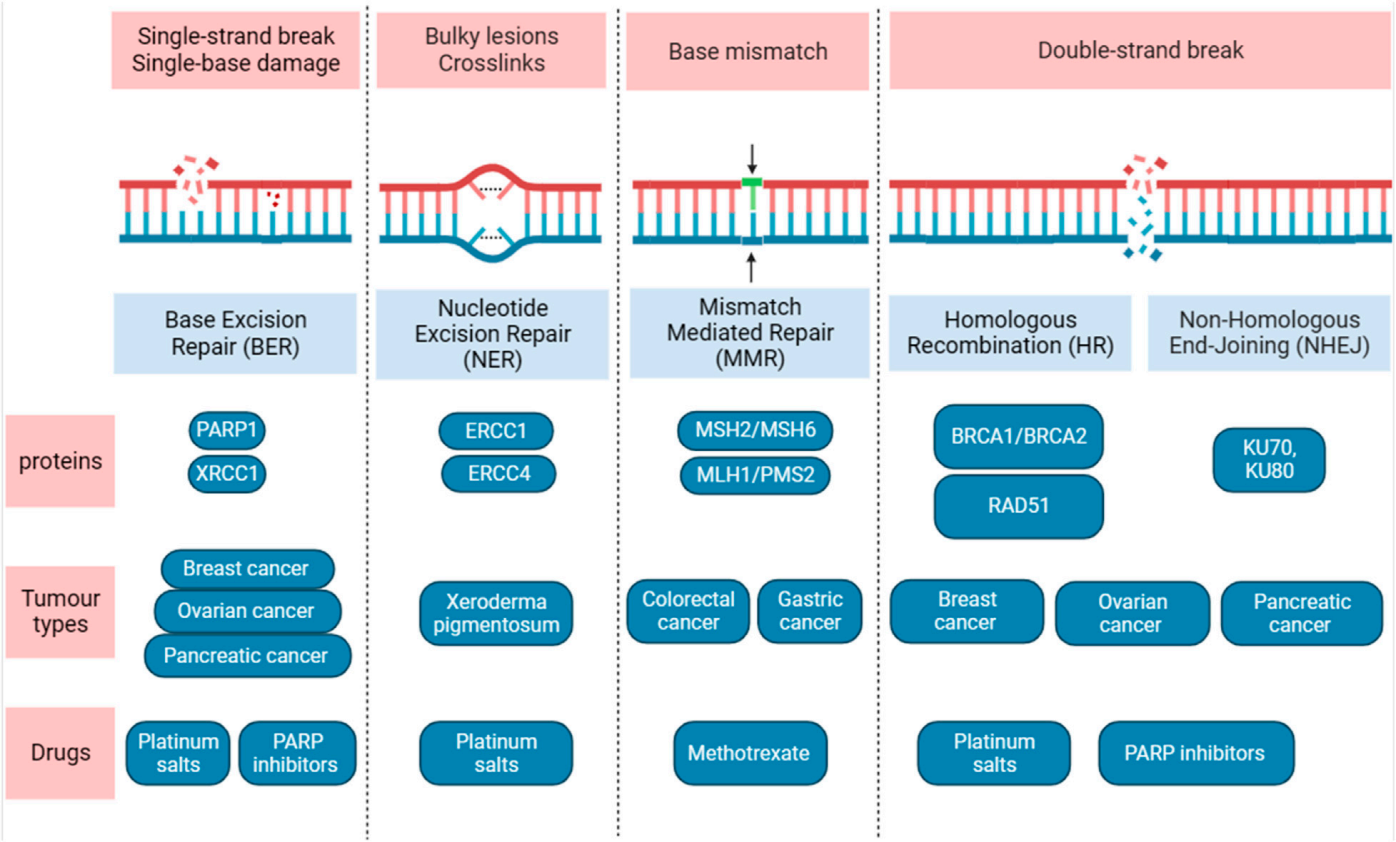
Overview of the five main DNA damage repair pathways. Diagram shows the DNA lesions caused by different sources and the DNA repair pathways. The major proteins, type of tumours, drugs used are shown. Figure is generated using Biorender.com.

The cell has the ability to recognize DNA damages and initiate DNA repair. Failure to do so can result in pathological disorders such as cancer. One of the hallmarks of cancer is the diversity of genetic and epigenetic mutations which is a characteristic of almost all types of cancers and provide a myriad set of resistance to chemotherapy drugs (Negrini et al., 2010). DNA damage is mainly repaired by five DNA repair pathways depending on the type of damage (Bernstein et al., 2002) (Figure 2).

The predominant repair responsible for the DNA bases damages is called base excision repair (BER). BER takes place in both nuclei and mitochondria and can protects against several diseases such as cancer, neurodegeneration and aging. It eliminates a short strand of DNA that contain the damaged base such. For example, oxidative damage to DNA that is caused by oxygen reactive species and DNA adduct which caused by interaction between segment of DNA with carcinogen agents such as cisplatin (Ank et al., 2006), acetaldehyde methylation and reactive oxygen species (Krieg, 2002). BER is initiated by a DNA glycosylases, and at least 12 distinct DNA glycosylases have been identified (Jacobs and Schär, 2012), each can recognize specific lesions. DNA glycosylases remove lesions base from DNA that generate apyrimidinic (AP) site which recruit PARP1. The major AP in human cells, also called APE1, is critical in protecting cells from toxic effects of DNA damage agents. Overexpression of AP in human has been linked to chemotherapy- and radiotherapy-resistance, thus several inhibitors of APE1 have been generated. One of the inhibitors that was identified called CRT0044876, and have been shown to inhibit BER pathway in solid tumor in an acidic tumor microenvironment resulting in oxidative DNA damage (Seo and Kinsella, 2009). Methoxyamine (MX) is also an inhibitor for AP and has been shown to increase the level of TMZ-induced DNA single strand breaks (Taverna et al., 2001). Interestingly, DNA glycosylases may play critical role in epigenetics, in previous studies, DNA glycosylase (TDG) knockouts display an embryonic lethal phenotype as TDG is believed to has critical epigenetic function in DNA methylations (Ermolaeva et al., 2013; Minko et al., 2020). Mismatch repair (MMR) is considered as excision-based repair system, and plays a critical role in repairing DNA replication errors and maintaining genomic stability (Classen et al., 2023). It has the ability to eliminate insertion or deletion loops and base-base mismatches that can occur during DNA synthesis. Germline mutations in the DNA MMR pair gene homologue hMLH1 has been linked with herited non-polyposis colon cancer (Morrison et al., 1994), also mutations in other MMR genes have been linked to sporadic colorectal (Poulogiannis et al., 2010) and gastric cancers (Leite et al., 2011). Nucleotide excision repair (NER) eliminate a single-stranded DNA molecule that contains short damaged 24–30 base pairs (Classen et al., 2023). It has a central role in recognizing lesions in double helix conformation which can be caused either by UV light or chemical agents that can give rise to DNA adducts. There are multiple steps as well as multiple proteins in the NER pathway that assemble at the damage sites (Isaacs et al., 1963). Individuals who were born with inherited mutation in NER pathway usually develop Xeroderma Pigmentosum (XP) syndrome and have high chances of developing tumors in skin with a median age of 8 years compared to 60 years in normal individuals (Ank et al., 2006). In addition, to overlapping symptoms associated with several disease such as cancer, immunological defects and developmental delay (Cleaver et al., 2009).

If repairing a single-strand lesions in DNA failed, double-strand break can occur which is the most dangerous type of DNA damage and can cause cell death. Moreover, inappropriate repair can lead to development of cancer or diseases that are associated with genomic instability. The two main pathways that are required to repair this type of break are non-homologous end-joining (NHEJ) and homologous recombination repair (HRR), each may compete for DSB repair (Rothkamm et al., 2003). Unlike HRR, the NHEJ does not require a DNA template to repair DNA lesions. Therefore, it is not restricted to a specific phase of the cell cycle, whereas HRR is only active when the a homologous template is available during the S and G2 phases of the cell cycle (Rothkamm et al., 2003; Mao et al., 2008). The NHEJ is mediated by a number of essential factors that are recruited to DSB sites. The first step in NHEJ is binding to the Ku70/Ku80 heterodimer (Ku) to the lesions DNA in DSB. Upon binding to DNA lesions, the Ku recruits the DNA-dependent protein kinase (DNA-PKcs) to generate the DNA-PK holoenzyme by which display kinase activity. In the HRR, the central player is a protein called Rad51 which function in all three phases of HR: pre-synapsis, synapsis and post-synapsis. Rad51 is loaded onto ssDNA in the pre-synapsis phase and during the synapsis, RAD51 regulate the formation of a physical connection between DNA substrate and homologous duplex DNA template. In the post-synapsis, when DNA is synthesized Rad51 dissociates from dsDNA to expose the 3′-OH required for DNA synthesis. Thus, targeting these DRR pathways can enhance response to immunotherapeutic agents (Classen et al., 2023; Hodson et al., 2023).

## 4 DNA-damage agent induces immune system activation

The first report to link the role of DNA in initiating immune signaling was reported in 1963 by Isaacs and colleagues (Isaacs et al., 1963). They showed that mouse cells stimulated with chick nucleic acid produced more cytokines and interferons (IFNs). Both bacterial DNA as well as RNA from viruses induce the expression of IFNs (Krieg, 2002; Ank et al., 2006). Similar to foreign DNA, damaged endogenous DNA has been shown to induce immune response. A study on the nematode *Caenorhabditis elegans* has shown innate immune response towards endogenous double-stranded breaks (DSBs) through initiating ERK1/2 MAPK signaling (Ermolaeva et al., 2013). The mechanisms of how DDR induces an immune response is still under investigation.

It is believed that DNA-damage agents can induces immunosuppression and eliminate different immune-cell populations as one of the most common toxicities of cytotoxic chemotherapy (Bellone et al., 2009; Kang et al., 2009). However, an increasing body of evidence now suggest that limiting the dose of DNA damage agent can also enhance tumor immunogenicity and shape the tumor microenvironment to enhance anti-tumor immunity (Bracci et al., 2014).

### 4.1 Upregulation of Ag-MHC complexes

Similar to all nucleated cells, tumor cells express MHC-I molecules on their surface where they present their endogenous antigens to cytotoxic CD8^+^ T cells; however, nearly 65%–90% of tumor cells have the ability to suppress the expression of Ag-MHC-I complex as a main mechanism of immune evasion (Garrido et al., 2016; Garrido and Aptsiauri, 2019). Mutation or deletion (hard lesions) of one of the MHC-I components such as the β2microglobulin (β2m; the gene is located on chromosome 15 in humans) and the MHC-I heavy chain (the gene is located on chromosome 6) or hypermethylation (soft lesion) of MHC-I and β2m genes may result in the loss of MHC-I on the cell surface (Garrido et al., 2010). In addition, ‘hard’ or ‘soft’ lesions in antigen-processing machinery (APM) components such as TAP (required for entry of peptides in the endoplasmic reticulum), immunoproteasome (No peptide) and other chaperon proteins can lead to the loss of MHC-I expression (Leone et al., 2013). The majority of MHC-I defects in human cancers is due to soft lesions while hard lesions are responsible for about 30%–40% of MHC-I deficient (Garrido et al., 2010). For example, 80% of Hodgkin lymphoma cells are MHC-I-deficient due to mutations of β2m (Reichel et al., 2015), and 75% of Diffuse Large B cell Lymphoma (DLBCL) show abnormal expression of β2m protein and were deficient in MHC-I expression (Challa-Malladi et al., 2011). Tumor cells at early stage are predominantly MHC-I positive but CTL target killing of MHC-I positive cells often induces a selective pressure for MHC-I negative tumor cells. Thus at later stages tumor cells often become MHC-I negative where they lack tumor infiltrating lymphocytes (TILs), a phenotype that is linked to poor patient outcome (Garrido et al., 2016). Inducing MHC-I expression is critical to increase the recognition of tumor cells by cytotoxic CD8^+^ T cells. MHC-I transfected malignant cells injected to syngeneic mice demonstrated oncogenicity that was associated with increased survival to tumor-bearing animals (Tanaka et al., 1985). Soft defects in MHC-I on tumor cells can be reversed using chemotherapy that is used to treat cancer. One of the strategies to restore MHC-I expression is by inducing epigenetic modifications using DNA methyltransferase (DNMT) inhibitors such as 5-azacytidine (Šímová et al., 2011) and histone deacetylase (HDAC) inhibitors such as entinostat (MS-275) and butyrate (Conte et al., 2018) (Table 1 for the FDA-approved drug to induce epigenetic modification to treat cancer). Epigenetic modification and immunotherapy can work synergistically to supress tumor mouse models (Šímová et al., 2011; Park et al., 2015). A study has shown that *in vivo* administration of 5-FU, cisplatin or SN-38 into the peritoneal cavity at low-dose induces expression of MHC-I in colorectal cancer cells, which suggest a potential mechanism to sensitize tumor cells to immunotherapy (Ohtsukasa et al., 2003). Chemotherapeutics drugs can also upregulate MHC-I in cancer cells. For example, tumor cells treated with gemcitabine and oxaliplatin have been shown to upregulate MHC-I expression and increase antigen presentation (Liu et al., 2010; Miyashita et al., 2017). Other drugs such as taxol, vinblastine and epothilone which act as microtubule destabilizers, enhanced MHC-I surface expression in a time dependent manner in ovarian cancer cells by increasing the production of cytokines such as IFNα, IL-1β, IL-6 and IL-12 (Pellicciotta et al., 2011). Furthermore, expression of HLA-B RNA is upregulated in ovarian cancer patients received paclitaxel-carboplatin (Peng et al., 2015), and drugs such as cyclophosphamide, gemcitabine and oxaliplatin enhances MHC-I expression in kidney, prostate, colon and breast cancer cells in a dose-dependent manner (Martins et al., 2009; Liu et al., 2010; Sistigu et al., 2014). Notably, not all chemotherapy drugs enhance MHC-I expression in all cancers. For instance, carboplatin failed to enhance MHC-I protein expression as well as RNA levels in ovarian cancer cell lines (Brunekreeft et al., 2020). Furthermore, depsipeptide FR901228, a histone deacetylase inhibitor, failed to elevate MHC-I surface protein expression in lymphoma, leukemia, cervical and breast cancer cell lines (Skov et al., 2005). Breast cancer tumors treated with topotecan (TPT) that targets topoisomerase I enhances MHC-I expression, and supernatant from TPT-treated breast cancer cells induces expression of cell-surface MHC-I in drug-naïve recipient cells. TPT-treated cells show increased secretion of interferon-β (IFN-β), TNF-α, IL-6 and IL-8 and activation of type I IFN signaling, which suggests role of cytokines and other secreted molecules produced upon treatment with TPT in inducing expression of MHC-I (Wan et al., 2012).

**TABLE 1:** FDA-approved drugs to induce epigenetic modification to treat cancer.

Type	Drug name	FDA-approved date	Tumor type	References
DNA methyltransferase inhibitors	Azacitidine (Vidaza)	May 2004	Myelodysplastic syndrome (MDS)	Issa et al. (2005)
	Decitabine (Dacogen)	May 2006	MDS	Saba (2007)
		April 2013	Acute Myeloid Leukemia (AML)	Malik and Cashen (2014)
	Guadecitabine (SGI-110)	November 2017	MDS	Daher-Reyes et al. (2019)
	Oral azacitidine (CC-486)	September 2020	Maintenance treatment of AML in patients who have achieved complete remission following induction therapy	Jen et al. (2022)
	Cytarabine/Decitabine Liposome (Vyxeos)	August 2017	Acute Myeloid Leukemia	Krauss et al. (2019)
Histone deacetylase inhibitors (HDACi)	Belinostat (Beleodaq)	July 2014	the treatment of relapsed or refractory peripheral T-cell lymphoma	Lee et al. (2015)
	Vorinostat (Zolinza)	October 2006	cutaneous T-cell lymphoma	Grant et al. (2007)
	Panobinostat (Farydak)	February 2015	multiple myeloma in combination with other drugs	Raedler (2016)
	Vorinostat (Zolinza)	October 2006	Cutaneous T-cell Lymphoma	Grant et al. (2007)
	Romidepsin (Istodax) a histone deacetylase (HDAC) inhibitor	November 2009	Peripheral T-cell Lymphoma	Barbarotta and Hurley (2015)
	Belinostat (Beleodaq) a histone deacetylase (HDAC) inhibitor	July 2014	Peripheral T-cell Lymphoma	Lee et al. (2015)
	Panobinostat (Farydak) histone deacetylase inhibitor	February 2015	Multiple Myeloma	Moore (2016)

### 4.2 Formation of neoantigen

Alexander *et al* (Alexandrov et al., 2013) have shown massive variance in the prevalence of mutations among tumors. These mutations may then result in creation of new epitopes that will be presented by MHC-I on the surface of tumor cells and recognized by T cells. The recognition of these epitopes will lead to activation of an anti-tumor response from the immune system. These epitopes are part of the so called neoantigens, which are true non-self antigens similar to viral or bacterial antigens. Tumors that fail to respond to immunotherapy have most likely less neoantigens regardless of the cancer type (Fang et al., 2022). Cancer cells that only express tumor associated antigens or overexpress normal molecules are also less likely to respond to immunotherapy since they do not activate anti-tumor immunity (Nagel et al., 2022). Interestingly, modest responses to PD-1/PD-L1 blockade in patients with non-small cell lung cancer (NSCLC) is strongly associated with weak formation of neoantigens (Anagnostou et al., 2017a). Therefore, enhancing neoantigen formation in tumors may sensitize them to checkpoint inhibitors.

Several studies have shown that low-dose of chemotherapy can induce the formation of neoantigen and therefore trigger anti-tumor immune response to target and kill tumor cells. For example, temozolomide can induce MMR defects in MMR-proficient cancer cells leading to increase in mutational load and expression of neoantigen and therefore enhanced tumor immune surveillance *in vivo* (Germano et al., 2017). These observations simply imply that increased mutational load may lead to increase neoantigen formation and, therefore, enhance immune surveillance. There is correlation between increased mutational load and increase antigenicity. Tumor cells are different in the amount of somatic mutation they carry, for example, lung and melanoma tumors have the highest mutational load (Alexandrov et al., 2013) and have the highest successful outcome when treated with immune checkpoint inhibitors (Carbognin et al., 2015). For example, high mutational load in melanoma and NSCLC is correlated with increased response to CTLA-4 and PD-1 blockades, respectively (Snyder et al., 2014; Rizvi et al., 2015a). A landmark phase II clinical trial showed that patients with colorectal cancer (CRC) or non-CRC cancers with MMR-deficient tumors treated with pembrolizumab had high objective response rates (ORRs) and significantly better progression-free survival (PFS) and overall survival (OS) compared to patients with proficient MMR (Le et al., 2015). There were 1782 somatic mutations on average observed in the MMR-deficient tumors whereas only 73 somatic mutations were identified in MMR-proficient tumors (Le et al., 2015). Furthermore, microsatellite unstable endometrial cancers with increased mutations in DNA polymerase epsilon (*POLE*) have high number of PD-1+ TILs which suggest increased antigenicity (Howitt et al., 2015). Similarly, glioblastomas–aggressive brain tumors unresponsive to anti-PD1 therapy–with mutation in DNA *POLE* have better response to pembrolizumab (Johanns et al., 2016). Tumors lacking mutation-associated neoantigens are resistant to immune checkpoint blockade in NSCLC patients (Anagnostou et al., 2017b). Furthermore, response to immune checkpoint blockade can be affected by intra-tumoral neoantigen heterogeneity with increased response in tumors with high neoantigen load (McGranahan et al., 2016). The FDA has approved pembrolizumab for patients with microsatellite instability-high (MSI-H) or MMR tumors in any type (Broderick, 2017). This is the first tissue agnostic drug approval by FDA that solely depends on the tumor genotype. Alteration in any DNA damage repair pathway can result in unique mutational signatures in tumors (Alexandrov et al., 2013). For instance, mutations in *BRCA1* or *BRCA2* genes in ovarian cancer cells have high number of clonal mutations and high neoantigen load (Strickland et al., 2016). High neoantigen load was associated with increased CD3^+^ and CD8^+^ TILs, enhance expression of PD-1 and PD-L1 in tumor-associated immune cells and improve overall survival (Strickland et al., 2016). Mutations in DNA repair genes like *BRCA2, POLD1*, *MSH2*, *POLE*, *PRKDC*, *RAD51C* and *RAD17* were associated with increase mutational load in NSCLC that were responsive to PD-1 blockade (Rizvi et al., 2015a). DNA damaging agents could raise the mutation burden and increase neoantigen formation. For example, treating *BRCA*-deficient tumor cells with PARP inhibitors result in accumulation of DNA lesions and increase genomic instability, which lead to tumor cell death (Farmer et al., 2005). However, cells that survive may have increased load of neoantigen (antigenicity) which can initiate T cell activation (Jackaman et al., 2012).

### 4.3 Immunogenic cell death (ICD)

DNA damaging drugs thought to induce cell death in an immunologically silent fashion, and this led to the neglecting of the role of the immune cells in enhancing the efficacy of chemotherapies. In addition to the guidelines set by biotech agencies to use immunodeficient mice to examine the effect of DNA damaging drugs on cancer cells (Johnson et al., 2001). Elimination of dead cells is a physiological event that plays a critical role in development (Galluzzi et al., 2015). This process was characterized based on membrane blebbing, chromatin condensation and morphological cell changes, and was thought to occur without an inflammatory response. However, new concepts have emerged stating that definition of cell death should not be characterized by morphological changes, but rather should include biochemical and metabolic changes (Galluzzi et al., 2012). This has led to the new definition of apoptotic cell death that releases antigens triggering immune responses prompted by dying cells (Radogna et al., 2019). What distinguishes DNA-damaging agents induced cell death from physiological-induced cell death, is the ability to generates dramatic changes in cell surface structures. Therefore, the release of soluble mediators that allow antigen presenting cells such as macrophages and dendritic cells to detect dying cells and then trigger anti-tumor immune response (Mezzapelle et al., 2021). Several DNA damaging agents such as bortezomib, oxaliplatin and anthracyclines have been shown to cause immunological cell death (Fucikova et al., 2011a; Michaud et al., 2011; Bezu et al., 2015). Cancer cells treated with anthracyclines show an increase in TLR3, Type I IFN and CXCL10 levels, which result in inhibiting tumor growth (Sistigu et al., 2014). In addition, many DNA-damaging agents such as mitoxantrone, oxaliplatin, and doxorubicin can induce ATP release from dying cancer cells (Kroemer et al., 2013). ATP is known for its role in metabolism signaling in cell and it can be released from the cells under physiological and pathological conditions such as plasma membrane rapture, mechanical stress and treatment with DNA damaging drugs. Once ATP is released from cells it can trigger immune response by activating inflammasome pathway and innate immune cells which lead to recruiting and priming of CD8^+^ T cells against tumor antigens (Ma et al., 2013).

In addition to exposure to neoantigens (antigenicity), activation of immune cells requires exposure to danger signals (adjuvanticity) (Galluzzi et al., 2017). Tumor cells can provide danger signal via damage-associated molecular patterns (DAMPs) which are released upon cell death. Release of DAMPs from dying or dead tumors recruit APCs to the site of immunogenic cell death (ICD) and initiate immune activation. The DAMPs released from ICD can be secretion of ATP, calreticulin (endoplasmic reticulum resident protein 60), double-stranded DNA, type I interferon and proinflammatory cytokines and secretion of CXCL10 (Kono and Rock, 2008). Not all DNA damaging drugs can induce same level of ICD, for example, cisplatin does not induce ICD as the same level as oxaliplatin as it cannot induce the release of calreticulin (Bezu et al., 2015). Any failure in inducing the release of DAMPs elements result in failure in inducing ICD as it is observed in cells treated with several DNA-damaging agents (Bezu et al., 2015).

### 4.4 Induce expression of NKG2D receptor’s ligands

The Natural Killer Group 2D (NKG2D) is an activation receptor expressed on NK cells, NKT cells and cytotoxic CD8^+^T cells (Liu et al., 2019). The pattern of receptor expression of NKG2D differ between species. In mice, almost all NK cells and activated CD8^+^ T cells exposed to viruses, intracellular bacteria and presumably other antigens express NKG2D while naive CD8^+^ T cells lack NKG2D expression (Duan et al., 2019). In human, almost all human peripheral blood naïve CD8^+^ T cells and cytotoxic CD8^+^ T cells express NKG2D and the level increases upon stimulation with interleukin-15 (IL-15) (Groh et al., 2001; Sutherland et al., 2002). NKG2D acts as co-stimulatory signal to enhance CD8^+^ T cells responsiveness against tumor cells *in vivo* and as stimulatory receptor on NK cells to bind to target cells. NKG2D was first discovered in genes screened for human natural killer (NK) cells along with NKG2A, NKG2C and NKG2E complementary DNAs (Houchins et al., 1990). These receptors are type-2 transmembrane receptors and are a member of the C-type lectin-like superfamily. Although they share similar name, NKG2D has different sequence compared to NKG2A, NKG2C and NKG2E which are all highly related in sequence (Houchins et al., 1990). NKG2D is homodimeric receptors which binds to several MHC class I-like cell-surface molecules, MICA, MICB, ULBP1-6 and DNAX Accessory Molecule-1 (DNAM-1) which can bind to PVR/CD155 and Nectin-2/CD112 belonging to the Ig-like superfamily (Cerboni et al., 2014), must of which are upregulated on stressed, infected and tumor cells. NKG2D ligands are a type of self-antigen and is a major co-stimulator of T cells (Das et al., 2001), NKG2D ligands can induce various biological effects in responding cells based on differences of their affinity for NKG2D. Signaling through NKG2D is required to stimulate NK cells and macrophages and co-stimulate CD8^+^ T cells to lyse tumor cell *in vitro* and to activate production of pro-inflammatory cytokines such as IFN-γ (Jamieson et al., 2002). Various pattern expression of NKG2D ligands are detected on different tumor cell lines and perforin-deficient mice developed RAE-1-expressed tumor cells (Smyth et al., 2005). This suggests that perforin was responsible for various expression of NKG2D ligand and tumor editing by the immune system could effect pattern expression of NKG2D ligands in tumors (Smyth et al., 2005).

NKG2D ligands are not detectable at the surface of normal cells, however many tumor cells and virus-infected cells significantly express NKG2D ligands. *Gasser et al*, have shown that NKG2D ligands expression in established tumor cell lines depends on the genotoxic stress (DiTullio et al., 2002). This finding supports the idea of the induction of NKG2D ligand expression by DNA damage. Several studies have shown that DNA-damaging agents and genotoxic stress induce expression of NKG2D ligands by activating a critical DNA damage checkpoint pathway induced by ATM or ART (Gasser et al., 2005). Upon treatment with reagents to induce intrinsic genome instability, tumor cells express MICA, MICB, ULBP1-6 which are ligands for activating immune receptor NKG2D (López-Larrea et al., 2008). Tumor cells exposed to DNA damaging agents induce most of NKG2D ligands such as Rae1, Mult1, and H60a genes in mice and the MICA and ULBP genes in humans (Gasser et al., 2005). Expression of DNAM-1 ligand was also increased upon DDR. Treatment with low doses of DNA-damage drugs induced the expression of NKG2D and DNAM-1 ligands in multiple myeloma (MM) cells through an ATM/ATR-dependent manner (Soriani et al., 2009). Temporary knockdown of ATM, ATR protein kinases or DNA damage checkpoint pathways such as Chk1 in tumor cells results in reduced expression of NKG2D ligands. Activation of ATM, ATR and the Chk1 is required for Ligand expression while suppression of ATR, ATM or Chk1 reduces ligand expression. Inhibition of ATM or Chk1 in the T2 cells, a murine ovarian epithelial cell line, reduces Rae1 levels at the cell surface (Gasser et al., 2005). However, further investigations need to be done to firmly support the role of DNA damage in NKG2D’s ligands expression.

### 4.5 STINGing the DNA can activate STING pathway

The Stimulator of Interferon Genes (STING) pathway is characterized by a mechanism which allows cells to sense foreign DNA released from viruses or microbes and also plays a critical role in sensing and detecting dying tumor cells (Barber, 2015). It is one of the immune mechanisms to recognize and target tumor cells through sensing damaged DNA (Barber, 2015). Damaged or foreign DNA can act as a DAMP to trigger innate immune system. It is detected by Cyclic guanosine monophosphate (GMP)–adenosine monophosphate (AMP) synthase (cGAS) as a DAMP and initiate type I IFNs and other cytokines signaling (Sun et al., 2013). To activate STING pathway, cGAMP synthase must interact with cytosolic DNA and catalyzes the synthesis of cGAMP, which acts as a second messenger to activate STING (Barber, 2015) and help to convert guanosine triphosphate (GTP) and ATP into the second messenger cyclic GMP-AMP (cGAMP) (Wu et al., 2013). cGAMP is a high-affinity ligand for STING and can induce transformational change to it (Wu et al., 2013). Once STING pathway is activated, it undergoes a conformation change that leads to its translocation from the endoplasmic reticulum (ER) to Golgi apparatus. This process helps to recruit and activate TANK-binding kinase 1 (TBK1) and IFN regulatory factor 3 (IRF3) to STING carboxyl terminus through a phosphorylation-dependent mechanism (Tanaka and Chen, 2012). Activated TBK1 phosphorylates IRF3, which will then relocate to the nucleus to result in transcription of type I interferon (IFN) genes (Chen et al., 2016). STING also activates NF-κB signaling pathway which leads to transcription of proinflammatory cytokines (See reference (Kato et al., 2017) for details on STING signaling pathway). In 2014, *Gajewski et al* (Woo et al., 2014) have shown that mice deficient in IRF3 or STING have clear defects in priming T cells and were unable to reject tumor cells. This data suggest that the activation of STING pathway is critical for antigen presenting cells to trigger T cell immunity against tumor cells and show that STING pathway is one of the innate immune sensing pathways to recognize tumor cells. Further evidences, suggest a role of STING in dendritic cells to sense circulating tumor DNA, engulf tumor cells and upregulate type I IFN production to trigger T cell immunity (Klarquist et al., 2014; Corrales et al., 2015).

Cancer cells expose to DNA-damaging agents can suffer a loss or alteration on DNA repair capacity, and this may contribute to activation of STING signaling pathway that is mediated by anti-tumor immunity. Upregulation of IFN signaling pathway mediated by STING signaling pathway has been shown in cells isolated from Atm-deficient mice as well as patients with Ataxia-Telangiectasia (AT) (Härtlova et al., 2015). Furthermore, activation of STING signaling pathway is observed following exposure to DNA-damaging agents. Genotoxic stress mediated by DNA-damaging agents induced a type I IFN response among a panel of breast cancer cell lines, and silencing the STING signaling pathway abolished this response (Gaston et al., 2016). Cytosolic DNA is key to activation of the STING signaling pathway, and *BRCA1/2*- as well as *ATM*-deficient cell lines have been shown to have increased levels of cytosolic DNA compared to wildtype cells (Härtlova et al., 2015; Parkes et al., 2017). Moreover, DNA-damaging drugs such as etoposide and cisplatin can increase cytosolic DNA (Ahn et al., 2014), the underlying mechanism of this cytosolic DNA increase upon exposure to DNA-damaging drugs is currently unknown.

Activation of the cGAS–STING pathway in APCs by cytosolic DNA can induce type I IFN signaling. Mice lacking STING fail to reject tumor cells and fail to respond to both radiation (Deng et al., 2014) and immune PD-L1 blockade (Wang et al., 2017b). Consistent with these finding, initiating cGAMP or STING using exogenous agonists can promote immunity and tumor rejection (Corrales et al., 2015; Demaria et al., 2015). Initiation of cGAS–STING pathway can improve the antitumor effect of chemotherapy and work synergistically with immune checkpoint inhibitors (Li et al., 2016; Wang et al., 2017b). The cGAS–STING pathway is active in some tumor type (An et al., 2019) and this activity can be increased by accumulation of genomic instability in tumor cells. For example, accumulated DNA lesions in prostate cancer induces STING-dependent type I IFNs resulting in tumor rejection (Ho et al., 2016). DNA lesions initiate cGAS–STING pathway in tumor cells leading to cell death which prevents tumorigenesis (Storozynsky and Hitt, 2020). cGAS–STING pathway links DNA damage to antitumor cellular response such as cell death and immune activation which make it an attractive pharmaceutical target for cancer therapy. Two phase I clinical trail (NCT03010176) (Harrington et al., 2018) and (NCT03172936) (Meric-Bernstam et al., 2023) are investigating the safety of using two sting agonists known as MK-1452 and MIW815 respectively. They are being tested in patients with lymphoma and advanced solid tumors in combination with KEYTRUDA, PD-1 inhibitor. Patients response to sting agonist as a monotherapy will proceed to receive the KEYTRUDA as combination.

### 4.6 Induced expression of IFNs

Lesions in DNA not only activate DNA repair system but also initiate multiple complex signaling pathways that promote cell proliferation, death and survival (Härtlova et al., 2015). The interferon (IFN) signaling pathway is an example of this complex. IFNs play a central role in regulating immune responses and are consist of three cytokine families, type I, II and III (Fensterl and Sen, 2009) and have a dual roles in innate and adaptive immunity. The type I IFN genes encode α and β, which is often activated in response to viral infections and induces innate immunity, type II IFN gene encodes (γ) which is induced following activation of T cell and NK cells, and type III IFN encodes (λ) which is primarily induced in response to bacterial and viral infections (Broggi et al., 2020).

The expression of IFN-β has been observed following exposure to DNA-damaging drugs such as Adriamycin, mitomycin C, etoposide and camptothecin (Brzostek-Racine et al., 2011). Tumor cells that are treated with DNA-damaging drugs stimulate IFN signaling *in vitro* and *in vivo* and produce more IFN-β. IFNs then increase DNA damage responses and promote immunity (Yu et al., 2015). DNA-damage drugs lead to increased expression of multiple IFN-stimulated genes, which lead to upregulation of type I IFNs enhancing anti-cancer immunity. DNA-damaging agents such as anthracyclines and oxaliplatin (Tesniere et al., 2010; Fucikova et al., 2011b) can induce ICD which activates antigen-specific T cells to secrete IFNγ, and initiates anti-tumor activation and promote tumor surveillance. Type I IFNs can also increase ICD, for example, treatment with anthracycline in various tumor cell lines induce production of type I IFNs and induce ICD (Sistigu et al., 2014). DNA lesions induce expression of IFN-α and IFN-λ-related genes. Chemotherapy can induce T-cell-mediated immune responses. Ovarian tumor cells treated with cisplatin or doxorubicin *in vitro* and then injected into mice show increase CD4^+^ T cell antitumor immunity with increase overall survival (Kim et al., 2012). Treatment efficacy in patients with platinum-resistant ovarian cancers increased significantly with low-doses of cisplatin and paclitaxel which induced a strong tumor-specific CD8^+^ T-cell response, via secretion of IL-2 and IFN-γ (Chang et al., 2012). Administration of 5-FU was reported to induce IFN-γ secretion by TILs and to eliminate myeloid-derived suppressor cells (MDSCs) *in vivo* resulting in increasing anti-tumor response (Vincent et al., 2010). In addition, Administration of both 5-FU and cisplatin recruits CD4^+^ and CD8^+^ T-cells to the tumor site in esophageal squamous cell carcinoma (Tsuchikawa et al., 2012).

The mechanism by which DNA-damaging drugs induce expression of IFNs was explained by *Fuchs et al* where they found that interferon regulatory factor 3 (IRF3) gets activated in an ATM-IKKα/β-dependent manner and increases the expression of IFN-β in response to double-stranded DNA breaks (Yu et al., 2015). IRFs and NF-kB are IFN-β enhancers. The IRFs are known to regulate IFN-stimulated genes including type I IFN genes and are known to have a multiple role in inducing immune response (Mogensen, 2019). Increased phosphorolation of IRF was observed in BRCA1/2-deficient cancer cells compared to BRCA1/2-positive cancer cells (Härtlova et al., 2015). DNA-damaging drugs induce double stranded DNA breaks that lead to the escape of free DNA from the nucleus in circulation, which trigger immune cells through pattern recognition receptors (Lind et al., 2022). Engagement of pattern recognition receptor lead to the activation of a cascade of signaling pathways, which include the IFN regulatory factors (IRFs) (Li and Wu, 2021). IRFs then act in inducing transcription of multiple genes, including type I IFN. The interaction of type I IFN with their receptors will trigger and enhance anti-cancer immunity response. Type I IFN has shown to increase tumor sensitivity to cisplatin which is known to be a weak inducer of ICD. Type I IFNs play a critical role in innate and adaptive immunity to promote anti-tumor response. It promotes survival of B cells, proliferation of CD8^+^ T cells and activation of DCs. It activates STING pathway after sensing DNA damage (Woo et al., 2014). Chronic exposure to type I IFNs can upregulate immune checkpoint receptors. Impairment of type I IFN signaling can attribute to acquired resistance to ICIs. Downregulation of IFN signaling reduces antigen presentation and hence limit activation of T cells. Patients treated with anti-PD-1 therapy for 6 months who relapsed have loss of function mutations in genes encoding JAK1 and JAK2 and loss of functional response to IFN-γ which were not present before treatment (Zaretsky et al., 2016). Defects in IFN-γ signaling-related genes including but not limited to JAK2, IRF1, IFNGR2, IFIT1/3, MTAP and miR31 have been detected in melanoma patients with anti-CTLA4 -resistance (Gao et al., 2016). Mutations in IFN-γ signaling-related genes have also been observed in anti-PD-1- resistance lung cancer (Gettinger et al., 2017). Mutations in overlapping genes between type I and type II IFN signaling pathways may suggest that Type I IFNs could play a role in resistance to ICIs. Cancer is usually associated with imbalance of Th1/Th2 immunity. DNA-damaging drugs such as cyclophosphamide (CTX) can restore this imbalance through induction of Th1-polarizing cytokines (IL-2 and IFN-γ) and supress induction of Th2 cytokines (IL-4 and IL-10) in mice (Bracci et al., 2007). Patients with advanced NSCLC treated with paclitaxel show a more robust anti-tumor immunity by increasing circulating IFN-γ-secreting CD8^+^ T-cells and IL-2-secreting CD4^+^T-cells therefore enhancing Th1 cellular immunity (Zhang et al., 2008). The nuclear kinase ataxia-telangiectasia mutated (ATM) is a critical transducer to response to DSBs. It belongs to the phosphatidylinositol 3-kinase (PI3K)-related kinase family which involve in the DNA-damage response. Transduction of DNA damage response signal by ATM occur via phosphorolation of checkpoint kinases Chk1 and Chk2, and the p53 tumor suppressor effectors. These effectors initiate cell cycle arrest to allow for DNA damage repair or promote apoptosis. During this time, activation of transcription factors can occur in response to DNA damage such as nuclear factor-kappa B (NF-κB) (Zhao et al., 2020). Activation of NF-κB induce the expression of cellular responses-related genes such as inflammation, proliferation and stress. It can also promote the induction of the IFN system, which is a well known anti-viral system.

## 5 DNA-damaging agents enhance response to immune checkpoint blockade

Immune checkpoint blockade implements the use of antibodies to target inhibitory signaling molecules on cancer and immune cells. The first immune checkpoint blockade to show a clinical benefit was the anti-CTLA-4 antibody ipilimumab in patients with metastatic melanoma (Hodi et al., 2010). Following the promising result of using anti-CTLA-4 blockade, evidence showed clinical benefit for using antibodies to target programmed death-1 (PD-1), clinically known as Nivolumab and Pembrolizumab (Hirano et al., 2005; Topalian et al., 2012) and its ligand (PD-L1) (Brahmer et al., 2012), available clinically as durvalumab, atezolizumab and avelumab (Pardoll, 2012). The use of these monoclonal antibodies to target immune checkpoints have been tested and approved in different type of diseases including advance melanoma (Mahoney et al., 2015; Raedler, 2015), non-small cell lung cancer (Rizvi et al., 2015b), and head and neck cancer (Forster and Devlin, 2018) with improving overall survival in metastatic settings. However, these drugs only benefit a minority of patients with cancer, and additional studies are required to investigate of combining these immunotherapeutic with other treatment modalities in different malignancies.

One of the tumor characteristics that may be more responsive to immune checkpoint blockade is the so-called “*immunologically hot*” tumors (Vareki, 2018). Hot tumors are the tumors that have been infiltrated with T cells, with an inflamed phenotype. Although T cell presence within the tumor is often a good prognostic biomarker, it is not enough to eliminate the tumor completely. As these tumors can put a brake on the T cells and therefore inhibit them to kill tumor cells. Hence, treatment with immune checkpoint blockade can eliminate these brakes from T cells and has shown a great successful rate in these kinds of tumors. Hot tumors often are associated with high mutational load, which could lead to increased DNA lesions and production of neoantigen on their surface. The expression of these antigens could make tumors more susceptible to immune cell recognition and hence enhances anti-tumor immunity. Examples of hot tumors are head and neck tumors, bladder tumor, NSCLC, and melanoma as well as tumors that have high microsatellite instability. In the other hand, cold tumors are tumor that have not generated strong immune response due to T cells being excluded within tumor cells. Since cold tumor cells have less T cell within it, immune checkpoint blockade is less likely to be beneficial compared to hot tumors. Tumors such as ovarian, prostate, and pancreatic tumors are challenging to response to immune checkpoint blockade as they consider cold tumors. Good news is, short-term treatment with chemotherapy could convert cold tumor into hot tumor and could modulate the immune response and increase sensitivity to immune checkpoint blockade (Heinhuis et al., 2019). Cancer with prediction to response to immune checkpoint blockade are those with higher mutation load. The same principle applied to tumor with increased genetic instability. Tumors with deficiency in microsatellite instability or MMR are more susceptible to response better to immune checkpoint blockade. Genetic instability could increase DNA lesions which lead to increase formation of neoantigens.

### 5.1 Sensitizing tumor to CTLA-4 blockade

Cytotoxic T-lymphocyte-associated antigen-4 (CTLA-4), also known as CD152, is a receptor expressed in T cells that undergo activation and play critical role in immune response. It belongs to immunoglobulin-related receptors that regulate T cell immunity and play inhibitory roles in T cell function. When antigen presenting cells (APC) such as Macrophages engulf dying cancer cells, it will present tumor epitope on their surface through MHC which will be then recognized by T-cell receptor (TCR) on T cells. This interaction will form signal one to activate T cells. However, this signal is not sufficient to generate T cell activation, as it needs a second signal which form when CD28 on T cell bind to B7 molecules on APC. CTLA-4 is homologues to CD28 with higher affinity to B7 molecules but conduct an inhibitory signal instead. CTLA-4 knockout mice have been shown to develop lethal lymphoproliferation (Waterhouse et al., 1995). Blockade of CTLA-4 lead to induce activation and proliferation of T cells and initiation of anti-tumor response. Tumor cells with high mutational load response better to CTLA-4 blockade. Melanoma patients, for example, have high response rate to anti-CTLA-4 due to high mutation burden in the tumor (Snyder et al., 2014). Hence increasing mutation load in tumor cells could sensitize tumor cells to CTLA-4 blockade. Ipilimumab, also known by its brand name as Yervoy, is a fully humanized monoclonal antibody that bind to CTLA-4 and prevent binding to B7 molecule and therefore block the inhibitory function of T-cell activation. It has been tested in advanced melanoma in clinical III trail and is an FDA-approved for the advanced metastatic melanoma. Furthermore, patients with NSCLC that received ipilimumab in combination with paclitaxel and carboplatin in randomized phase II trial had longer immune-related progression-free survival (PFS) (Lynch et al., 2012).

Gemcitabine is a nucleoside analogue that widely used to treat ovarian, lung, breast and other type of cancer. However, this treatment is usually limited by the inhibitory T cell molecule CTLA-4 and therefore combining gemcitabine with CTLA-4 blockade may improve patient outcome. A study (Lesterhuis et al., 2013) has used two type of non-immunogenic mouse tumor and treat them with both gemcitabine and anti-CTLA blockade result in better immune response and tumor regression with long-term protective immunity. This data show that combining chemotherapy with immune checkpoint inhibitor may have synergistic effect in treating cancer. Furthermore, Treatment with CTLA-4 blockade alone was not sufficient in supressing tumor growth in MOPC-315 tumor (Mokyr et al., 1998). However, when combine with low dose of chemotherapeutic drug, melphalan, the growth of tumor was significantly reduced. The authors (Mokyr et al., 1998), also showed that anti-CTLA-4 blockade showed improved anti-tumor cytotoxicity only from splenocytes isolated from melphalan-treated -tumor bearing mice. These data suggest that the success outcome of CTLA-4 blockade treatment is significantly improved when combine with DNA-damage agents to improve immunogenic microenvironment of the tumor.

### 5.2 Upregulation of PD-1/PD-L1

**FIGURE 3:**
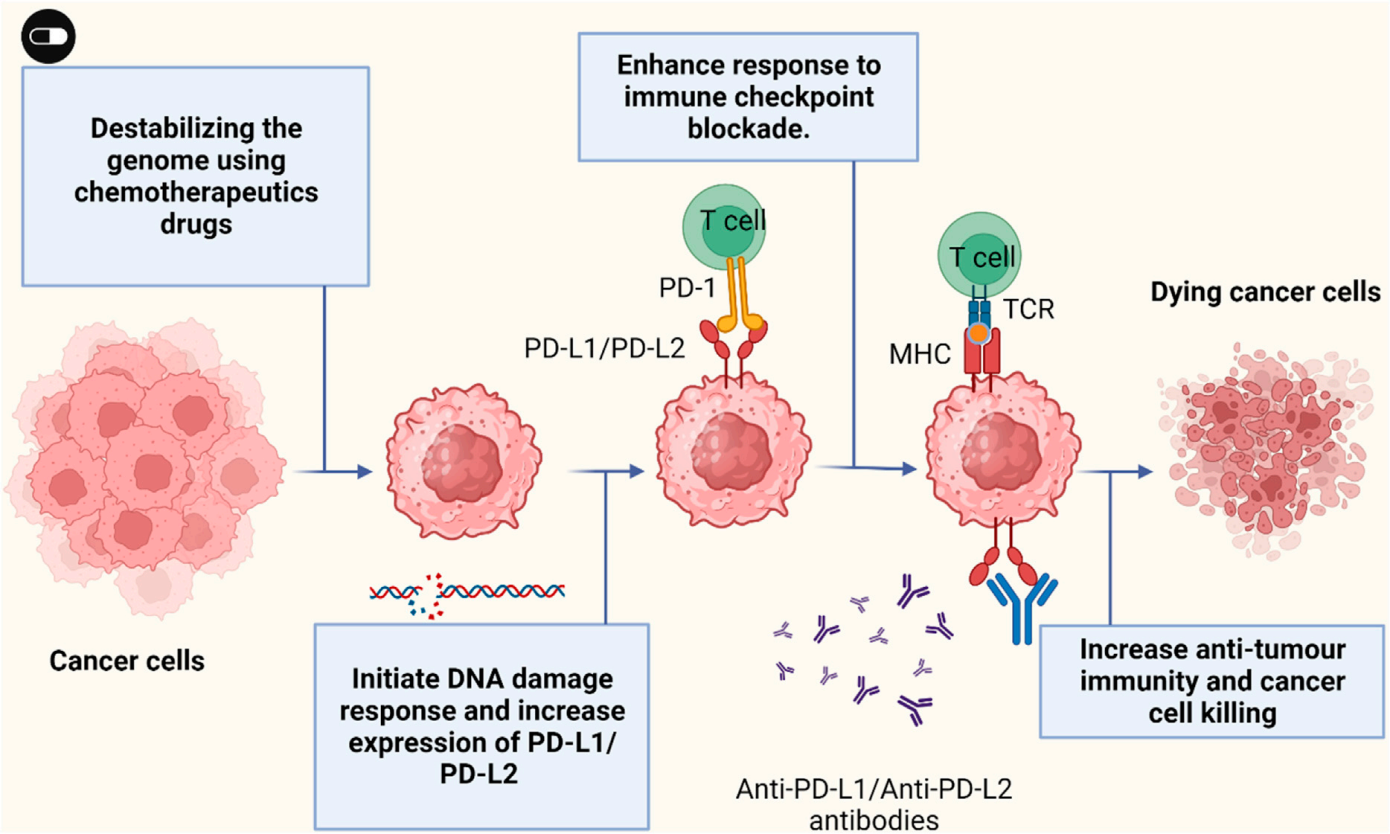
Cancer cells exposed to chemotherapeutic drugs can increase PD-L1/PD-L2 surface expression and sensitize cancer cells to immune anti-PD-L1/anti-PD-L2 therapy. Figure is generated using Biorender.com.

PD-L1 surface expression is dynamic and can be affected by multiple factors such as the type of DNA damaging agent and tumor type (Figure 3). Tumor cells exposed to cytotoxic drugs such as cisplatin or paclitaxel upregulate PD-L1 expression (Grabosch et al., 2015). An *in vivo* treatment of mice harbour aggressive 2F8 ovarian tumor with cisplatin show increase expression of PD-L1 on cell surface and respond better to PD-L1 blockade with increase survival (Grabosch et al., 2015). Cisplatin also could increase PD-L1 expression in hepatoma H22 cells and could also activate ERK1/2 phosphorolation which suggest that PD-L1 expression is dependent of phosphorolation of ERK1/2 (Qin et al., 2010). Breast cancer cells treated with etoposide and paclitaxel upregulated PD-L1 surface expression (Majidi et al., 2021). In Leukemia cells, PD-L1 and PD-1 expression was significantly increased with decitabine treatment (Yang et al., 2014). These data suggest that use of PD-1/PD-L1 blockade after chemotherapy as a strategy to enhance patient outcome. Blockade of PD-1/PD-L1 was successful in melanoma and NSCLC which characterise by high mutation load that cause neoantigen formation triggered by chemotherapy (Borghaei et al., 2015). Notably, losing these neoantigen lead to acquired resistance to anti-PD-1 therapy in NSCLC patients (Anagnostou et al., 2017b). Cancer cells such as melanoma and non-small cell lung carcinoma with high mutation load respond better to PD-1 blockade. Moreover, patients carrying tumor with microsatellite instability have excellent response to anti-PD-1 blockade (Le et al., 2015). In this study, patients were given pembrolizumab, the anti-PD-1 antibody, and their respond to treatment was significantly associated with the mutation load of tumor (Le et al., 2015). The United States Food and Drug Administration (FDA) has approved anti-PD-1 blockade treatment of metastatic tumors with microsatellite instability-high or MMR-deficiency.

### 5.3 Upregulation of Fas/FasL

Induction of T cell apoptosis by cytokines such as tumor necrosis factor (TNF) family makes them a good target to enhance cancer immunotherapy. Fas ligand (FasL) is a member of TNF and is a 40-kDa type II transmembrane protein that binds to Fas also known as CD95 (Yajima et al., 2019), which is a 45–52-kDa glycosylated cell surface protein. Many cancer cell express Fas and FasL in relatively high level (Ryan et al., 2006; Peshes Yaloz et al., 2007), suggesting that tumor cells would be sensitive to Fas-induce apoptosis. Several lines of evidences have suggested that DNA-damage agents can induce expression of FasL, which will bind to Fas and initiate the death receptor pathway. In hepatoma cells (Xia et al., 2017), neuroblastoma cells (Galenkamp et al., 2015), T cell leukemia (Nyakern et al., 2006), and other tumor cell types (Fulda et al., 1998). The FasL mRNA was increase after treatment with different DNA damage drugs. These drugs include, doxorubicin, methotrexate (Friesen et al., 1996; Friesen et al., 1997), etoposide, teniposide (Fulda et al., 1997; Kasibhatla et al., 1998), cytarabine (Friesen et al., 1997), cisplatin (Fulda et al., 1997) and bleomycin (Müller et al., 1997). The level of FasL protein has been increased after many of these treatments (Friesen et al., 1996; Friesen et al., 1997), and, the level of Fas receptor increased after treatment (Müller et al., 1997). A study has shown (Peng et al., 2001) that 5FU induced upregulation of FasL which lead to resistance of colon cancer cells to 5FU drug. The involvement of Fas/FasL pathway in 5FU-induced apoptosis points out the possibilities of new therapeutic opportunities. These data suggest that Fas/FasL can be upregulated after treatment with DNA damage drug. This pro-apoptotic role of Fas/FasL signaling pathway made a promising targeting for anti-cancer immunotherapy, therefore Fas/FasL blockade could be a good target to enhance patient outcome.

Although Fas/FasL signaling is critical in inducing tumor apoptosis, Both Fas and FasL have an important role in depleting peripheral T cell population. Blockade of Fas/FasL in T cells could reduce T cell apoptosis and enhance anti-tumor immunity. However, blockade of Fas/FasL could trigger lethal events and Intravenous administration of anti-Fas blockade caused lethal hepatitis in mice (Ogasawara et al., 1993). Systemic delivery of anti-FasL blockade associate with greater risk of lethal damage to the liver in human while. This toxic side effect can be avoided by local delivery of anti-Fas/anti-FasL blockade instead of systemic delivery. Rensing-Ehl *et al* shows that local delivery of anti-FasL blockade eliminate T cell apoptosis efficiently without systemic toxicity and without inducing lethal damage to the liver (Rensing Ehl et al., 1995).

## 6 Systematic review of the clinical evidence for combination of DNA repair blockade and immune checkpoint blockade

**FIGURE 4:**
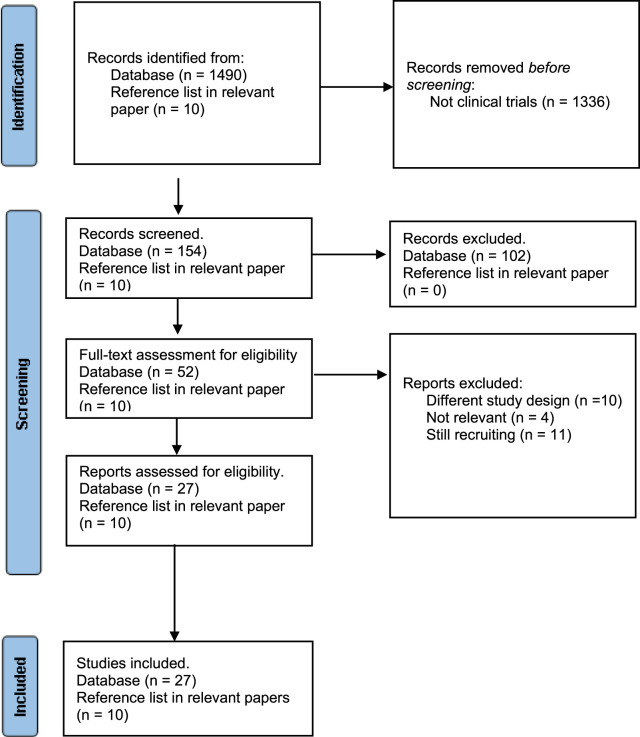
Flow chart of included and excluded studies using PRISMA 2020 flow diagram for new systematic reviews (Page et al., 2021b).

There has been an increased number of clinical trials that focused on combining DNA-damage drug with immune checkpoint blockade and illustrate their effect on patient’s outcome. In total, 164 clinical trials were retrieved through databases and reference list of related papers. After applying the inclusive criteria, a total of 37 clinical trials were included in the study. A flow chart of the study screening and selection process is shown in (Figure 4A, B). No date restriction was applied to the search. The majority of the articles were published in the last 5 years which reflect the rapid focus on inducing DNA damage in combination with ICI to improve treatment outcome. Risk bias assessment is reported in (Supplementary Figure S1). Pre-clinical studies have shown that therapeutic resistance is the main problem in many tumors. It is proposed that CD8^+^ T cells, also known as cytotoxic lymphocytes (CTLs), have the ability to overcome drug-resistance tumor cells. But their efficacy may be supressed by the tumor microenvironment or by cytotoxic-induced cell death. Therefore, introducing high-frequency low-dose, known as metronomic chemotherapy, could enhance the ability of CTLs to eliminate drug-resistance tumor by providing tolerated stimulation for CTLs without inducing T cells death. In addition, supressing tumor cells to stop their prognosis. Combining immune checkpoint inhibitors with the metronomic chemotherapy drug can further enhance anti-tumor immune response. Clinical trials investigating the effect of combining both DNA-damage agent with immune checkpoint blockade have significantly increased and is described below and in (Table 2).

**TABLE 2:** Clinical trials investigating the effect of combining both DNA-damage agent with immune checkpoint blockade.

Clinical trial	Cancer type	Combination	Stage	Outcome in combination therapy
Skin cancer				
NCT00324155 (Robert et al., 2011)	Untreated Metastatic Melanoma	Ipilimumab plus Dacarbazine	Phase 3	Improved OS
NCT03071406 (Kim et al., 2022)	Advanced Merkel cell carcinoma	Nivolumab and ipilimumab with or without stereotactic body radiation therapy	Phase 2	No improvement
Lung cancer				
NCT00527735 (Reck et al., 2012)	Extensive-disease-SCLC	Ipilimumab plus paclitaxel and carboplatin	Phase 2	Phased ipilimumab, but not concurrent ipilimumab, improved irPFS.
IMpower133 (Horn et al., 2018)	Extensive-stage SCLC	Atezolizumab plus carboplatin and etoposide	Phase 3	Improved OS and PFS
KEYNOTE-189 (Gandhi et al., 2018)	Metastatic NSCLC	Pembrolizumab plus pemetrexed and platinum-based chemotherapy	Phase 3	Improved OS and PFS
KEYNOTE-021 (Awad et al., 2021)	Metastatic non- Nonsquamous NSCLC	Pembrolizumab plus pemetrexed and carboplatin	Phase 1b/2	Improved ORR and PFS
CheckMate 9LA (Paz-Ares et al., 2021)	Advanced NSCLC	Nivolumab plus ipilimumab and chemotherapy	Phase 3	Improved OS and PFS
NCT02621398 (Jabbour et al., 2020)	Stage II-IIIB NSCLC	Pembrolizumab, Paclitaxel, Carboplatin, and Radiation Therapy	Phase 1	Improved PFS
NCT02572843 (Rothschild et al., 2021)	NSCLC	Durvalumab plus Neoadjuvant Chemotherapy	Phase 2	One-year event-free survival rate of 73%
NCT02888743 (Schoenfeld et al., 2022)	NSCLC	Durvalumab plus tremelimumab alone or in combination with low-dose or hypofractionated radiotherapy	phase 2	Radiotherapy did not increase responses to combined PD-L1 plus CTLA-4 inhibition in patients with NSCLC resistant to PD(L)-1 therapy
GEMSTONE-301 (Zhou et al., 2022)	Advanced, unresectable, stage III NSCLC	Sugemalimab versus placebo after concurrent or sequential chemoradiotherapy	phase 3	Sugemalimab after definitive concurrent or sequential chemoradiotherapy could be an effective consolidation therapy for patients with stage III NSCLC whose disease has not progressed after sequential or concurrent chemoradiotherapy
Taniguchi et al. (2022)	Previously Treated Advanced or Recurrent ICI-Naïve NSCLC	Nivolumab plus Docetaxel	phase 3	Improved OS and PFS
BTCRC-LUN15-029 (Salous et al., 2023)	Advanced NSCLC previously treated with a PD-1 or PD-L1 inhibitor	Chemotherapy plus pembrolizumab	Phase 2	Improved PFS
The PROLUNG (Arrieta et al., 2020)	Previously Treated Advanced NSCLC	Pembrolizumab Plus Docetaxel	Phase 2	Improved ORR and PFS in patients with advanced NSCLC
NCT02937116 (Jiang et al., 2021)	metastatic nonsquamous or squamous NSCLC	sintilimab in combination with chemotherapy	phase 1b	Sintilimab plus chemotherapy exhibited manageable toxicity and an encouraging antitumor activity in patients with nsqNSCLC and sqNSCLC
Gastrointestinal cancer				
KEYNOTE-590 (Sun et al., 2021)	Locally advanced or metastatic esophageal or gastroesophageal junction carcinoma	Pembrolizumab plus 5-fluorouracil and cisplatin	Phase 3	Improved OS and PFS
KEYNOTE-062 (Shitara et al., 2020)	Advanced gastric or gastroesophageal junction adenocarcinoma	Pembrolizumab plus fluorouracil and cisplatin or capecitabine	Phase 3	No statistically significant improvement in overall survival compared to chemotherapy alone
CA 209-678 (Tai et al., 2021)	Advanced HCC	Radioembolisation with Y90-resin microspheres followed by nivolumab	Phase 2	Improved ORR
NCT03122509 (Segal et al., 2021)	Mismatch Repair-proficient Metastatic Colorectal Cancer	Durvalumab and Tremelimumab with Concurrent Radiotherapy	Phase 2	Did not meet the prespecified endpoint criteria
CheckMate649 (Janjigian et al., 2021)	Advanced gastric, gastro-oesophageal junction, and oesophageal adenocarcinoma	Nivolumab plus oxaliplatin and capecitabine or leucovorin, fluorouracil, and oxaliplatin	Phase 3	Improved OS and PFS
NCT03143153 (Doki et al., 2022)	Advanced Esophageal Squamous-Cell Carcinoma	Nivolumab plus chemotherapy	phase 3	Both first-line treatment with nivolumab plus chemotherapy and first-line treatment with nivolumab plus ipilimumab resulted in significantly longer overall survival than chemotherapy alone
KEYNOTE-966 (Kelley et al., 2023)	Advanced biliary tract cancer	Pembrolizumab in combination with gemcitabine and cisplatin	phase 3	Improved OS
CheckPAC (Chen et al., 2022)	Refractory Metastatic Pancreatic Cancer	Nivolumab With or Without Ipilimumab Combined With SBRT	Phase 2	Antitumor activity and favorable safety profiles were demonstrated after treatment with SBRT/nivolumab/ipilimumab
Xie et al. (2020)	Metastatic PDAC	Durvalumab plus SBRT	Phase 2	Demonstrates a modest treatment benefit in patients with metastatic PDAC.
Bladder cancer				
KEYNOTE-361 (Powles et al., 2021)	Metastatic urothelial cancer	Pembrolizumab plus gemcitabine and cisplatin or carboplatin	Phase 3	No statistically significant improvement in PFS
IMvigor130 (Galsky et al., 2020)	Metastatic urothelial cancer	Atezolizumab plus gemcitabine and carboplatin or cisplatin	Phase 3	Improved PFS
Blood cancer				
NCT04541277 (Gao et al., 2023)	Relapsed/refractory acute myeloid leukemia	PD-1 inhibitor combined with DNA hypomethylation agent + CAG regimen	phase 2	Improved outcomes in r/r AML patients with lower pretherapy leukemia burden. irAEs were mild and low-grade
NCT02961101 and NCT03250962 (Liu et al., 2021)	Relapsed/refractory Hodgkin lymphoma	Camrelizumab plus decitabine	Phase 2	Improved PFS
Breast, ovarian and prostate Cancer				
NCT02819518 (Cortes et al., 2022)	Advanced Triple-Negative Breast Cancer	Pembrolizumab plus Chemotherapy	phase 3	Improved OS
The Neo-PATH (Ahn et al., 2022)	ERBB2-Positive Stage II/III Breast Cancer	Neoadjuvant Pertuzumab, Atezolizumab, Docetaxel, and Trastuzumab Regimen	Phase 2	An acceptable pCR rate and modest toxic effects
IMpassion130 (Schmid et al., 2018)	Metastatic triple-negative breast cancer	Atezolizumab plus nab-paclitaxel	Phase 3	Improved PFS
NCT03430479 (Takada et al., 2022)	Bone metastasis in patients with HER2-negative metastatic breast cancer	Nivolumab combined with palliative radiation therapy	Phase Ib/II	Palliative RT combined with nivolumab was safe and showed modest anti-tumor activity
JAVELIN Ovarian 100 (Monk et al., 2021)	Previously untreated epithelial ovarian cancer	Chemotherapy with or without avelumab followed by avelumab maintenance	Phase 3	Results do not support the use of avelumab in the frontline treatment setting
NCT02484404 (Karzai et al., 2018)	Metastatic castration-resistant prostate cancer	Durvalumab plus Olaparib	Phase 1/2	Demonstrates efficacy, particularly in men with DDR abnormalities
Head and neck cancer				
NeoTGP01 (Huang et al., 2022)	Resectable locally advanced head and neck squamous cell carcinoma	Neoadjuvant toripalimab combined with gemcitabine and cisplatin	phase Ib	Achieves promising pathological complete response
KCSG HN17-11 (Jung et al., 2022)	Recurrent or Metastatic Nasopharyngeal Carcinoma	Nivolumab plus Gemcitabine	Phase 2	Nivolumab plus gemcitabine showed promising efficacy with favorable toxicity profiles in patients with advanced NPC in whom platinum-based combination chemotherapy failed
Paediatric cancer				
NCT2813135 (Pasqualini et al., 2021)	Paediatric relapsed/refractory solid tumours	Nivolumab and metronomic cyclophosphamide	Phase 2	Well tolerated but had limited activity in this paediatric setting

Immune-related progression-free survival (irPFS). Progression-free survival (PFS). Overall survival (OS). Objective response rate (ORR). Non-small cell lung cancer (NSCLC). Pancreatic Ductal Adenocarcinoma (PDAC). Stereotactic Body Radiotherapy (SBRT).

### 6.1 Skin cancer

The first combination that were evaluated were done with ipilimumab, the anti-CTLA-4, and dacarbazine, patients with untreated metastatic melanoma were giving dacarbazine with ipilimumab or dacarbazine with placebo (Robert et al., 2011). Interestingly, patients receiving ipilimumab in addition to dacarbazine have shown significant increase in overall survival compared to dacarbazine alone. Moreover, patients receiving combined treatment have remarkable enhanced in overall survivor with a minimum of 3 years of follow-up (20.8% compared to 12.2%) (Robert et al., 2011). Furthermore, a phase 2 clinical trial examined the efficacy of combined nivolumab and ipilimumab with stereotactic body radiotherapy to treat 50 patients with advanced Merkel cell carcinoma (Kim et al., 2022). The combination of Nivolumab and ipilimumab shows high ORR in ICI-naïve patients and those previously treated with ICI. However, additional treatment with stereotactic body radiotherapy did not improve ORR significantly in the combined treatment (Kim et al., 2022).

### 6.2 Lung cancer

In randomize trial involving patients with untreated metastatic non-small lung cancer aimed to investigate the effect of combining two of DNA-damage agents with ipilimumab (Reck et al., 2012). 204 patients received carboplatin with paclitaxel plus ipilimumab in a phased treatment schedule, had greater overall survival compare to carboplatin with paclitaxel alone (Reck et al., 2012). Moreover, 616 patients were enrolled in double-blind phase 3 clinical trial examined the combination of pembrolizumab with standard chemotherapy for first-line treatment of metastatic NSCLC without targetable EGFR/ALK mutations (Gandhi et al., 2018). The combination group had significantly improved overall survival after 1 year follow-up compared to placebo combination. A phase 3, double-blind, placebo-controlled trial evaluated the combination of atezolizumab with carboplatin and etoposide in patients with extensive-stage SCLC (Horn et al., 2018). The result shows significant improvement in OS and PFS in patients receiving combination therapy compared to chemotherapy alone (Horn et al., 2018). The additional of durvalumab to neoadjuvant chemotherapy was evaluated for resectable stage IIIA (N2) NSCLC patients (Rothschild et al., 2021). Patients received three cycles of cisplatin and docetaxel followed by two doses of durvalumab for up to 1 year post surgery. The radiographic response rate improved from 43% after chemotherapy to 58% after sequential immunotherapy and major pathologic response achieved in 62% of the resected patients. The one-year event-free survival (EFS) rate was 73% in the combinational therapy which suggest that combining durvalumab to neoadjuvant chemotherapy is effective and safe. Destabilizing the genome using radiotherapy was tested in combination with durvalumab (anti-PD-L1) and tremelimumab (anti-CTLA-4) in patients with resistant to anti-PD-1 and anti-PD-L1-targeted therapy (Schoenfeld et al., 2022). The trial enrolled 90 patients to receive either durvalumab and tremelimumab alone or in combination with low-dose or hypofractionated radiotherapy. The study was conducted for 1 year or until disease progression. However, the radiotherapy did not improve response to durvalumab and tremelimumab in patients resistant to anti-PD-1 and anti-PD-L1-targeted therapy (Schoenfeld et al., 2022).

### 6.3 Gastrointestinal cancer

The efficacy of combining pembrolizumab plus chemotherapy as a first-line treatment for advanced oesophageal cancer and Siewert type 1 gastro-oesophageal junction cancer patients was tested (Sun et al., 2021). This phase 3, double-blind, randomized, placebo-controlled trial was conducted in 26 countries across 168 medical centers. The results showed that combination therapy was superior to chemotherapy alone in patients with oesophageal squamous cell carcinoma and PD-L1 combined positive score (CPS) of 10 or more (Sun et al., 2021). This suggests combination of pembrolizumab plus chemotherapy for the treatment of patients with previously untreated, advanced oesophageal squamous cell carcinoma. Furthermore, a phase 3, open-label trial examined the effectiveness of combining nivolumab plus chemotherapy or nivolumab plus the ipilimumab for advanced esophageal squamous-cell carcinoma (Doki et al., 2022). The trial enrolled 970 patients with unresectable advanced, untreated, recurrent, or metastatic esophageal squamous-cell carcinoma. The patients were randomly assigned at a ratio of 1:1:1 to receive nivolumab plus chemotherapy, nivolumab plus ipilimumab, or chemotherapy alone. The result shows that both combination of nivolumab plus chemotherapy and nivolumab plus ipilimumab resulted in improved OS compared to chemotherapy alone. This result was observed in patients with tumour PD-L1 expression of 1% or greater as well as in the overall population. Improved PFS was also found in patients treated with nivolumab plus chemotherapy compared to patients treated with chemotherapy alone. The improvement in PFS was not seen in patients treated with nivolumab plus ipilimumab compared to patients treated with chemotherapy alone (Doki et al., 2022). This study suggests the potential therapy of both nivolumab plus chemotherapy as first-line treatments for advanced esophageal squamous-cell carcinoma.

In another hand, some of the clinical trials show no superior effect of combination therapy. For example, phase three randomized controlled trial, combination of pembrolizumab and chemotherapy were examined to treat 763 patients with advanced gastric/gastroesophageal junction cancer (Shitara et al., 2020). Patients with PD-L1 combined positive score ≥1 were randomized 1:1:1 to the three arms (pembrolizumab alone, pembrolizumab plus chemotherapy, or chemotherapy alone). Combination therapy did not improve overall or PFS compared to chemotherapy alone.

### 6.4 Bladder cancer

The effects of atezolizumab, with platinum-based chemotherapy was evaluated in patients with metastatic urothelial carcinoma in IMvigor130 phase 3 trial (Galsky et al., 2020). The study enrolled 1,213 patients for a period of 2 years from 221 centers in 35 countries. The patients were randomly assigned to receive atezolizumab plus platinum-based chemotherapy (group A), atezolizumab alone (group B), or platinum-based chemotherapy and placebo (group C). In the group A and C, patients received with 21-day cycles of gemcitabine in addition to carboplatin or cisplatin on day one of each cycle with either atezolizumab or placebo. In group B, patients received atezolizumab on day one of each 21-day cycle. The results found the addition of atezolizumab to platinum-based chemotherapy improved PFS in patients compared to platinum-based chemotherapy (Galsky et al., 2020). This suggests the combination of atezolizumab plus platinum-based chemotherapy as potential therapy for first-line treatment for metastatic urothelial carcinoma. Another phase three clinical trial tested combination of pembrolizumab with chemotherapy (gemcitabine and cisplatin or carboplatin) in patients advanced urothelial carcinoma (Powles et al., 2021). The overall survival was similar between single treatment group and combination group.

### 6.5 Blood cancer

Patients with relapsed/refractory (r/r) acute myeloid leukemia (AML) response poorly to Anti-PD-1 blockade despite the higher PD-1 and PD-L1 expression. A single-arm phase 2 study evaluated the efficacy of combining PD-1 inhibitor with a DNA hypomethylating agent (HMA) + CAG to improve overall response in patients who had failed previous therapy (Gao et al., 2023). Total of 27 patients were enrolled in the study and the ORR was 63% with various levels of remission observed. The median OS and EFS were 9.7 and 9.2 months respectively. Among the responders, the median OS was not reached while among the non-responders it was 2.4 months (*p* = 0.002) (Gao et al., 2023). The combination therapy showed improved outcome particularly in patients with a lower pretherapy leukemia burden (Gao et al., 2023). In another study conducted on patients with relapsed/refractory classical Hodgkin lymphoma (cHL), the combination of the anti-PD-1 agent camrelizumab and the DNA demethylating agent was evaluated (Liu et al., 2021). Total number of 61 patients were enrolled in randomized phase 2. The result showed improved PFS compared to camrelizumab alone and complete remission rate of 79% in the patients treated with combination therapy compared to 32% in the patients treated with camrelizumab alone. The median PFS was 35 months compared to 15.5 months in combination group and camrelizumab group, respectively. Furthermore, the study found that lower tumour burden, female gender and less previous therapies were good prognostic factors for durable remission with camrelizumab therapy. Meanwhile, in the combination group, improved PFS was observed in patients with larger tumor burdens and those with prior therapies (Liu et al., 2021).

### 6.6 Breast, ovarian and prostate cancer

A phase three clinical trial demonstrated clinical improvement with the addition of atezolizumab and nab-paclitaxel compared to placebo plus nab-paclitaxel in untreated metastatic triple-negative breast (TNB) cancer patients (Schmid et al., 2018). Furthermore, pembrolizumab plus the chemotherapy (nanoparticle albumin-bound paclitaxel, paclitaxel, or gemcitabine–carboplatin) was also tested in patients with previously untreated locally recurrent inoperable or metastatic TNB cancer (Cortes et al., 2022). The phase 2 trial enrolled 847 patients and randomly assigned them to receive pembrolizumab–chemotherapy or placebo–chemotherapy (Cortes et al., 2022). The total follow-up median was 44.1 months. In the pembrolizumab–chemotherapy group, the median overall survival was 23.0 months and in the placebo–chemotherapy group was 16.1 months (Cortes et al., 2022). This result support the use of pembrolizumab–chemotherapy combination to improve OS in patients with advanced TNB cancer. In epithelial ovarian cancer patients, platinum-based chemotherapy shows good response, however, about 70% will relapse within 3 years of treatment. To evaluate the efficacy of avelumab maintenance and avelumab combination treatments to enhance response, an open-label, randomised, phase 3 trial was conducted (Monk et al., 2021). Total of 998 women aged 18 years and older with stage III–IV epithelial ovarian, fallopian tube, or peritoneal cancer were enrolled from 159 centres in 25 countries. The total follow-up median for PFS was 10.8 months, For the avelumab maintenance group, it was 11.1 months; for the avelumab combination group, it was 11.0 months; and for the control group, it was 10.2 months. This results do not support the use of combinational therapy as a frontline treatment setting.

Two different PARP inhibitors (PARPi), olaparib and talazoparib, upregulate PD-L1 expression on cancer cells, which in turn, reduces PARPis efficacy. Hence, targeting PD-L1 can restore anti-tumor immunity and enhance the antitumor activity of PARPis (Jiao et al., 2017). This observation led to numerous clinical trails investigation the combination of PD-L1 blockade with PARPis. The phase I/II clinical trail (NCT02484404) showed olaparib in combination with durvalumab, an anti-PD-L1 antibody, to be an effective combination for metastatic castration-resistant prostate patients (Karzai et al., 2018). Olaparib was assessed at 300 mg orally every 12 h and durvalumab at 1,500 mg i. v every 28 days until disease progression or intolerable toxicity. Only two out of seventeen patients have developed tolerated toxicity. Median radiographic progression-free survival (rPFS) is 16.1 months best results were seen in patients with mutation in DDR genes and with fewer peripheral myeloid-derived suppressor cells.

### 6.7 Head and neck cancer

Treatment with ICI for the advanced head and neck squamous cell carcinoma (HNSCC) has shown promising treatment outcomes for patients. However, further studies are needed to advance the treatment outcomes and overcome resistance. In an open label, single-arm, phase Ib clinical trial, the neoadjuvant toripalimab combined with gemcitabine and cisplatin was evaluated in 23 patients with advanced HNSCC (Huang et al., 2022). The radiographic response rates were 5.0% for complete response (CR), 40.0% for partial response (PR), and 55.0% for stable disease (SD). The ORR was 45.0% in addition to increase level of CD4, CD8, CD20, and CD38-positive cell in the tumour after neoadjuvant chemotherapy (Huang et al., 2022). In another phase 2 trial, the combination of nivolumab plus gemcitabine was evaluated in patients with recurrent or metastatic nasopharyngeal carcinoma (Jung et al., 2022). Patients received nivolumab and gemcitabine every 2 weeks until disease progression or intolerable toxicity. Among the 36 patients, the disease control rate was 97.2% and ORR was 36.1% and the OS rate at 6 months was 97.0% (Jung et al., 2022).

### 6.8 Pediatric cancer

In an European multicenter phase 2 clinical trial, the safety and activity of nivolumab in combination with oral cyclophosphamide ± irradiation in 13 pediatric patients was assessed (Pasqualini et al., 2021). The main histologies were neuroblastoma, high-grade glioma, and desmoplastic small round cell tumour (DSRCT). The safety profile of the combination therapy was similar to the nivolumab alone. Lymphocytopenia was reported with the treatment of cyclophosphamide ± irradiation. Tumour samples revealed modest intratumour CD3^+^ T-cell infiltration and low mutational load with an immunosuppressive tumour microenvironment. The combination therapy did induce enhance modulation of circulating T cells however, neutrophil-to-lymphocyte ratio (NLR) did increase. The study concluded that treatment with the combination was well tolerated in paediatric patients with relapsed/refractory solid tumours however, the anti-tumour activity was limited (Pasqualini et al., 2021).

These studies have established a clear contribution of the DNA-damage agent as an active agent with immune checkpoint blockade. This contribution offers patients with the advanced stage of the disease the benefit and long-term survival rate. Moreover, these results established the idea that activating the immune cells with immune checkpoint blockade after treatment with DNA-damage drug can provide a remarkable benefit effect.

## 7 Discussion

Pre-clinical studies and clinical trials have investigated and validated the benefit of destabilizing the genome using DNA repair inhibitors to activate the anti-tumor immunity and sensitize the tumor to immune checkpoint blockade. The concept of inhibiting DNA repair pathways in cancer treatment is becoming deeply investigated to enhance immunotherapy in cancer patients, as we are entering an era where genetic mutations that are responsible for specific kind of tumor will help to guide in selecting the targeted drug. The aim of using DNA repair inhibitors is to exploit tumor lesions or mutations in DNA repair pathways and to generate spontaneous fetal replication lesions. This kind of treatment is highly advanced compared to current chemotherapies as it can minimize the side effects of toxic drugs while at the same time increase the level of toxic lesions that should lead to target cancer cells. Immunotherapies are currently investigated in the clinical trials and some of the immune checkpoint blockades are already approved in treating multiples cancer diseases and is currently investigated further for numerous types of cancer. Even though immune checkpoint blockade shows beneficial results, several patients both within or across cancer type develop resistance to the treatment. Therefore, identifying prognostic biomarkers is critical step to overcome resistance.

Several studies have suggested that genomic instability represent an important predictive biomarker for immune checkpoint blockade response and suggested the use of DNA-damage agents in combination with immune checkpoint blockade as a treatment strategy. The Keynote-164 clinical trail shows the benefit of using pembrolizumab in patients with advanced MSI-H colorectal cancer (Le et al., 2018). This support the fact that tumor with deficient in MMR response better to immune checkpoint blockade. Further clinical trails are currently investigating the response rate in other DNA-repair-deficient to further support this strategy. The principle in most of these clinical trails is the concept that inducing genomic instability often results in generating spontaneous fetal replication lesions which leads to increase mutation load and formation of neoantigen. This have been shown to correlate with increase response to immune checkpoint blockade in numerous of cancer patients. However, this correlation is more complex and the association between DNA-damage agents and immune checkpoint blockade need further investigations. Although the majority of the clinical trials showed promising approach in destabilizing the genome with ICI to improved overall survival, few studies showed no impact compared to single therapy. This could be due to several reasons. Three of the clinical trials (Segal et al., 2021; Kim et al., 2022; Schoenfeld et al., 2022) used radiotherapy as an approach to destabilize the genome in combination with ICI, all of which showed limited anti-tumour activity compared to single agent therapy. Unlike the systematic effect of chemotherapies, radiotherapy is a local treatment which can not kill tumour cells that spread to another part of the body. Furthermore, tumour cell sensitivity to radiotherapy may influence the combination therapy outcome. Whereas with chemotherapies, there are different option of drugs that can work on different type of cancer which makes it more effective to treat wide range of cancers.

It is critical to understand that while DNA-damage agents could lead to improve the beneficial outcome of using immune checkpoint blockade, it also comes with potential downsides. These agents lack specificity and can affect healthy cells leading to wide range of side effects such as nausea, anemia, organ damage, impairment of immune system and sometime secondary cancers.

## 8 Conclusion and future prospects

Understanding how DNA-damage agent enhances immune system could lead to new discoveries in generating strategic treatment for cancer patients. Moreover, many exogenous agents that can target genes or proteins in DNA repair mechanisms as well as genomes that are critical in DNA repair need to be identified. Unlike *BRCA1-*and *BRCA2-*deficient tumors, most tumors are not well defined in DNA repair defect, therefore it is challenging to identify which gene of the DNA repair to target. The critical challenge here is how to maintain the level of genomic instability during the course of treatment. It is likely that answering this question will reveal more genetic questions and perhaps lead to the need of more therapeutic approaches.
