# Supplementary figures and images for: Destabilizing the genome as a therapeutic strategy to enhance response to immune checkpoint blockade: a systematic review of clinical trials evidence from solid and hematological tumors
Source: Front Pharmacol. 2024 Jan 9;14:1280591. doi: 10.3389/fphar.2023.1280591 (PMC10803447; doi:10.3389/fphar.2023.1280591)

Supplementary figure 1:


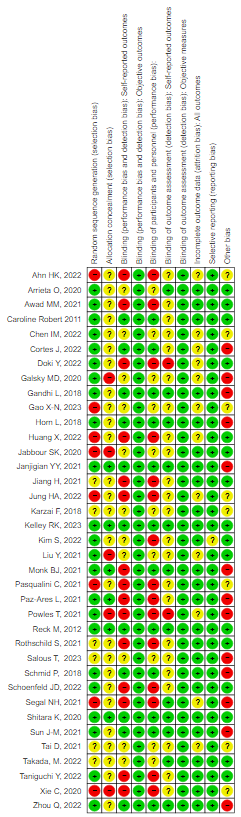


1. Risk of bias summary


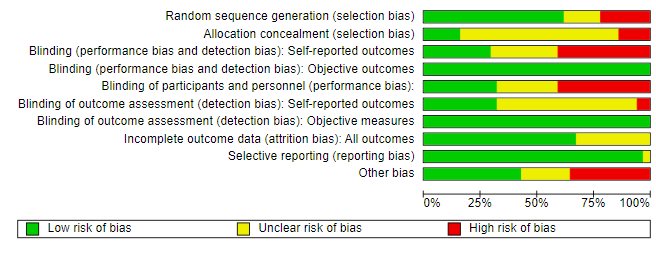


1. Risk of bias graph

Supplement: Supplementary file 1 [file DataSheet1.docx]
